# Supplementary material for: Dynamic prediction model of spontaneous combustion risk in goaf based on improved CRITIC-G2-TOPSIS method and its application
Source: PLoS One. 2021 Oct 27;16(10):e0257499. doi: 10.1371/journal.pone.0257499 (PMC8550420; doi:10.1371/journal.pone.0257499)
Supplement: S1 File — (DOCX) [file pone.0257499.s001.docx]

I'm here to clarify there are no legal or ethical restrictions, I have uploaded all the data required for the paper as a Supporting Information file, and data is always available. If you have any other requirements, please let me know by email, and I am very happy to reply to you. I look forward to hearing from you very much. Best wishes to you!

In addition to the data already provided in this paper, other data are as follows：

1. Taking the Shanxi Jinniu Coal Mine as an example. Working face 1303 is located at level 1030 in mining coal seam 9. The coal seam is 6.17-m thick and has a slope of 8°–14° (average of 10°), making it a grade II spontaneous combustion coal seam. Its coal dust explosion index is 45.79%, i.e., explosive.

2. Prediction and evaluation system for spontaneous combustion hazards：

| Spontaneous goaf combustion risks *U*_i_ | Single index |
| --- | --- |
| spontaneous combustion tendency *U*_1_ | degree of coal metamorphism *U*_11_ |
|  | rate of CO temperature increase per unit *U*_12_ |
|  | temperature difference of coal oxidation-reduction ignition point *U*_13_ |
|  | coal oxygen inhalation capability *U*_14_ |
| air leakage and oxygen supply conditions *U*_2_ | thickness of coal seam *U*_21_ |
|  | depth of coal seam *U*_22_ |
|  | geological structure *U*_23_ |
|  | slope of coal seam *U*_24_ |
| heat accumulation and emission conditions *U*_3_ | air leakage strength *U*_31_ |
|  | temperature of surrounding rock *U*_32_ |
|  | air leakage time *U*_33_ |
| goaf conditions and specifications *U*_4_ | residual coal thickness *U*_41_ |
|  | advance speed *U*_42_ |
|  | goaf area *U*_43_ |
|  | air supply *U*_44_ |

3. Evaluation grades and value ranges for spontaneous goaf combustion：

| Grade | I | II | III | IV | V |
| --- | --- | --- | --- | --- | --- |
| Value range | (90,100] | (70,90] | (60,70] | (40,60] | (0,40] |

4. Expert scores and improved indicator scores：

| Single index | Expert evaluation | First evaluation | Second evaluation | Third evaluation |
| --- | --- | --- | --- | --- |
| degree of coal metamorphism *U*’ 11 | (0.4,0.6) | 70 | 80 | 60 |
| rate of increase of CO temperature *U’* 12 | (0.8,1.0) | 50 | 70 | 50 |
| temperature difference of coal oxidation-reduction ignition point *U*’ 13 | (0.7,0.9) | 60 | 80 | 70 |
| coal oxygen absorption capacity *U*’ 14 | (0.6,0.8) | 50 | 60 | 60 |
| thickness of coal seam *U*’ 21 | (0.5,0.7) | 30 | 40 | 30 |
| depth of coal seam *U*’ 22 | (0.6,0.8) | 50 | 90 | 80 |
| geological structure *U*’ 23 | (0.3,0.5) | 60 | 80 | 70 |
| slope of coal seam *U*’ 24 | (0.4,0.6) | 70 | 90 | 80 |
| air leakage strength *U*’ 31 | (0.7,1.0) | 60 | 80 | 70 |
| temperature of surrounding rock *U*’ 32 | (0.7,0.9) | 50 | 80 | 60 |
| air leakage time *U*’ 33 | (0.7,0.9) | 80 | 70 | 80 |
| residual coal thickness *U*’ 41 | (0.3,0.5) | 50 | 60 | 55 |
| advance speed *U*’ 42 | (0.7,0.8) | 60 | 80 | 70 |
| goaf area *U*’ 43 | (0.4,0.6) | 60 | 60 | 80 |
| air supply *U*’ 44 | (0.5,0.7) | 60 | 70 | 65 |

5. Weights of the evaluation indexes：

| Index | *U*’ 11 | *U*’ 12 | *U*’ 13 | *U*’ 14 | *U*’ 21 | *U*’ 22 | *U*’ 23 | *U*’ 24 |
| --- | --- | --- | --- | --- | --- | --- | --- | --- |
| Weight | 0.052 | 0.090 | 0.081 | 0.071 | 0.062 | 0.071 | 0.043 | 0.052 |
| Index | *U*’ 31 | *U*’ 32 | *U*’ 33 | *U*’ 41 | *U*’ 42 | *U*’ 43 | *U*’ 44 | --- |
| Weight | 0.088 | 0.081 | 0.081 | 0.043 | 0.073 | 0.052 | 0.062 | --- |

6. Update indicators and dynamic weights of each indicator：

| Index | Dynamic evaluation | | | $\Delta q$ | | | Renewed factors   | | | Standard weight | | | Dynamic weight | | | Average weight |
| --- | --- | --- | --- | --- | --- | --- | --- | --- | --- | --- | --- | --- | --- | --- | --- | --- |
|  | 1 | 2 | 3 | 1 | 2 | 3 | 1 | 2 | 3 | 1 | 2 | 3 | 1 | 2 | 3 |  |
| *U*’ 11 | 74 | 80 | 60 | 4 | 0 | 0 | 5 | 1 | 1 | 0.0635 | 0.0568 | 0.0474 | 0.123 | 0.027 | 0.0203 | 0.0568 |
| *U*’ 12 | 53 | 70 | 50 | 3 | 0 | 0 | 4 | 1 | 1 | 0.0785 | 0.086 | 0.0684 | 01216 | 0.041 | 0.0293 | 0.064 |
| *U*’ 13 | 62 | 78 | 68 | 2 | 2 | 2 | 3 | 3 | 3 | 0.0847 | 0.0885 | 0.0837 | 0.0984 | 0.126 | 0.1077 | 0.1107 |
| *U*’ 14 | 50 | 62 | 63 | 0 | 2 | 3 | 1 | 3 | 4 | 0.0619 | 0.0581 | 0.068 | 0.024 | 0.083 | 0.1167 | 0.0746 |
| *U*’ 21 | 30 | 41 | 32 | 0 | 1 | 2 | 1 | 2 | 3 | 0.0324 | 0.0339 | 0.0301 | 0.0125 | 0.0323 | 0.0387 | 0.0278 |
| *U*’ 22 | 50 | 89 | 80 | 0 | 1 | 0 | 1 | 2 | 1 | 0.0619 | 0.0873 | 0.0863 | 0.024 | 0.0831 | 0.037 | 0.048 |
| *U*’ 23 | 62 | 81 | 69 | 2 | 1 | 1 | 3 | 2 | 2 | 0.045 | 0.047 | 0.0451 | 0.0523 | 0.0447 | 0.0387 | 0.0452 |
| *U*’ 24 | 71 | 90 | 81 | 1 | 0 | 1 | 2 | 1 | 2 | 0.0635 | 0.0639 | 0.064 | 0.0271 | 0.0304 | 0.0549 | 0.0375 |
| *U*’ 31 | 60 | 80 | 70 | 0 | 0 | 0 | 1 | 1 | 1 | 0.0921 | 0.0961 | 0.0936 | 0.0357 | 0.0457 | 0.0402 | 0.0405 |
| *U*’ 32 | 52 | 82 | 63 | 2 | 2 | 3 | 3 | 3 | 4 | 0.0706 | 0.0885 | 0.0775 | 0.082 | 0.1263 | 0.133 | 0.1138 |
| *U*’ 33 | 83 | 73 | 82 | 3 | 3 | 2 | 4 | 4 | 3 | 0.113 | 0.0774 | 0.1009 | 0.1751 | 0.1473 | 0.13 | 0.1508 |
| *U*’ 41 | 48 | 62 | 54 | 2 | 2 | 1 | 3 | 3 | 2 | 0.0375 | 0.0352 | 0.0353 | 0.0436 | 0.0502 | 0.0303 | 0.0414 |
| *U*’ 42 | 59 | 80 | 71 | 1 | 0 | 1 | 2 | 1 | 2 | 0.0764 | 0.0797 | 0.0787 | 0.0592 | 0.0379 | 0.0675 | 0.0549 |
| *U*’ 43 | 60 | 61 | 78 | 0 | 1 | 2 | 1 | 2 | 3 | 0.0544 | 0.0425 | 0.0616 | 0.0211 | 0.0404 | 0.0793 | 0.047 |
| *U*’ 44 | 63 | 68 | 63 | 3 | 2 | 2 | 4 | 3 | 3 | 0.0649 | 0.0592 | 0.0593 | 0.1005 | 0.0845 | 0.0763 | 0.0871 |
